# Western Criticism

**Published:** 1899-05

**Authors:** 


### WESTERN CRITICISM.

It is pleasant to observe that our friends in the West have taken to repeated critical observations of men and things in the East. It is always a sign of healthy progress when one turns upon his neighbor to find the "mote," for sooner or later he will discover the "beam" somewhere in his own mental anatomy. There is hope, therefore, for the editor of the *Western Dental Journal,* and his readers may look forward joyfully to the time when his journal will present "expensive cuts" for their edification and instruction in art. In a recent number the editor and his assistant were suddenly awakened to the fact that the "witch-fires didn't do much for Salem, after all." This fact should have a wide circulation, for the impression has long been held that the goblins of the long ago have had much to do with the rush of travel to that interesting old town for many years past, and which will likely be continued for years to come.

The witchery that so disturbed our trade contemporary lies in the fact that one of Salem's good citizens, and a dentist, read a paper before the American Academy of Dental Science in Boston, and in due time it found its way upon our pages. In order that he and his assistant may not fail in receiving due notice in that cultivated quarter of the republic, the editorial and addition are quoted in full.

"THE MERIAM EXTENSION CROWN.

"The initiative original article in the INTERNATIONAL DENTAL JOURNAL for March describes the method of H. C. Meriam, of Salem, Mass., in applying the loss of a first bicuspid, which reminds us of specimens of prehistoric dentistry. The method is advised where the first molar has been lost and the second molar has taken its place. A band is fitted around the molar,—which he says can be done 'without any trimming,' as 'the second superior molar often tapers slightly, being a little larger at the neck than at the crown' (*sic*). An English tube tooth is selected for the missing bicuspid, and after banding is connected with the molar band, by a bar of spring gold wire, curved around the intervening bicuspid, 'leaving it free and clean.' This contraption is then cemented in place. The article is illustrated by an expensive cut, showing a bicuspid supplied on each side; on one side the bicuspid is entirely gone, and on the other the root remains and the new tooth is allowed to rest upon it (though why the root did not entirely support the crown, goodness knows).

"On account of the prominence of the article and the expensive cut (by the way, the cut shows that upon the right central root is a poorly adjusted crown), we suppose *some one* must have thought this kind of bridge a good thing. Our opinion is that, under ordinary circumstances, it might last forty-eight hours, that the piece would be filthy if it did last longer, and the molar holding the band and the bicuspid 'left free and clean' would be ruined. In the discussion following the description of this case, Dr. Meriam said, in speaking of a similar case, 'When I had sent my modest bill, the check came back with a note which said, "If you had charged me one thousand dollars, I should have called it cheap."' Oh, dear! oh, dear! the witch-fires didn't do much for Salem, after all."

The language made use of in the following, while it may not suit the æsthetic tastes of the city honored by the name of Hawthorne, is worthy of reproduction.

"*Editor Western Dental Journal:*

"Permit me to refer you to the first article in the INTERNATIONAL DENTAL JOURNAL for March, by Dr. H. C. Meriam, of Salem, Mass., on 'Extension Crowns,' and say, in reference to it, that for innate folly, ignorance, and superstition old Salem still holds the palm. Verily, Mr. Editor, we hold to the old, old truth, 'The wise men came from the East,' and never went back. Beggar that I am, I am ever poor in speech to express how utterly damnable is every statement of the author. No wonder bridge-work is in disrepute among Eastern patients.

"C. L. HUNGERFORD, D.D.S."

It may interest the editor of the *Western Dental Journal* and his collaborator to be informed that the editor of this journal was quite aware that the method presented was not entirely novel; indeed, aside from cementing the band over the molar, almost precisely the same plan was used in the insertion of a single tooth worn by himself over thirty years ago.

It is probably useless to argue with our critics that such a fixture has a certain value, and may be made as permanent and as cleanly, even more so, than the permanent abomination bridges so much in vogue in certain quarters, where malodorous conditions are apparently not regarded as of much consequence.

When dentists who write for journals will spend as much time preparing artistic models as the author of "The Meriam Extension Crown," they will find editors more than willing to secure the very best ability attainable to have them properly illustrated. It has been very rare in our editorial experience that a more beautifully prepared model has been sent to this office, and it is worthy of special commendation that in one instance, at least, it was demonstrated that the writer of the article was equal to the occasion. When dentists learn to prepare their manuscripts properly and, where cuts are needed, draw the models, or have them drawn, with some degree of artistic skill, editors of dental journals will treat more kindly requests for illustrations.

No doubt our brother in Kansas City will agree with this as a general proposition, and should he have occasion to need added art work on his journal we can cordially recommend the eastern cities where the printers' and engravers' art is understood and appreciated.

